# Fat oxidation at rest and during exercise in male monozygotic twins

**DOI:** 10.1007/s00421-019-04247-x

**Published:** 2019-10-31

**Authors:** Jari E. Karppinen, Mirva Rottensteiner, Petri Wiklund, Kaisa Hämäläinen, Eija K. Laakkonen, Jaakko Kaprio, Heikki Kainulainen, Urho M. Kujala

**Affiliations:** 1 Faculty of Sport and Health Sciences, University of Jyväskylä, Jyväskylä, Finland; 2 Gerontology Research Center, Faculty of Sport and Health Sciences, University of Jyväskylä, Jyväskylä, Finland; 3 Department of Medicine, Central Finland Health Care District, Jyväskylä, Finland; 4 Exercise Translational Medicine Center and Shanghai Center for Systems Biomedicine, Shanghai Jiao Tong University, Shanghai, China; 5 Department of Epidemiology and Biostatistics, Centre for Environment and Health, School of Public Health, Imperial College London, London, UK; 6 FirstBeat Technologies Ltd, Jyväskylä, Finland; 7 Department of Public Health, University of Helsinki, Helsinki, Finland; 8 Institute for Molecular Medicine Finland, University of Helsinki, Helsinki, Finland

**Keywords:** Twins, Exercise, Lipid metabolism, Oral glucose tolerance

## Abstract

**Purpose:**

We aimed to investigate if hereditary factors, leisure-time physical activity (LTPA) and metabolic health interact with resting fat oxidation (RFO) and peak fat oxidation (PFO) during ergometer cycling.

**Methods:**

We recruited 23 male monozygotic twin pairs (aged 32–37 years) and determined their RFO and PFO with indirect calorimetry for 21 and 19 twin pairs and for 43 and 41 twin individuals, respectively. Using physical activity interviews and the Baecke questionnaire, we identified 10 twin pairs as LTPA discordant for the past 3 years. Of the twin pairs, 8 pairs participated in both RFO and PFO measurements, and 2 pairs participated in either of the measurements. We quantified the participants’ metabolic health with a 2-h oral glucose tolerance test.

**Results:**

Fat oxidation within co-twins was correlated at rest [intraclass correlation coefficient (ICC) = 0.54, 95% confidence interval (CI) 0.15–0.78] and during exercise (ICC = 0.67, 95% CI 0.33–0.86). The LTPA-discordant pairs had no pairwise differences in RFO or PFO. In the twin individual-based analysis, PFO was positively correlated with the past 12-month LTPA (*r* = 0.26, *p* = 0.034) and the Baecke score (*r* = 0.40, *p* = 0.022) and negatively correlated with the area under the curve of insulin (*r* = − 0.42, *p* = 0.015) and glucose (*r* = − 0.31, *p* = 0.050) during the oral glucose tolerance test.

**Conclusions:**

Hereditary factors were more important than LTPA for determining fat oxidation at rest and during exercise. Additionally, PFO, but not RFO, was associated with better metabolic health.

## Introduction

Fat oxidation rates at rest (Goedecke et al. 2000; Robinson et al. 2016) and during exercise (Venables et al. 2005; Randell et al. 2016; Fletcher et al. 2017) vary among individuals. The key determinants of resting fat oxidation (RFO) are not precisely identified in the scientific literature. During exercise, the main determinant of substrate use is exercise intensity (Romijn et al. 1993). The peak fat oxidation (PFO) rate is usually achieved at moderate exercise intensities (~ 40–60% of maximal oxygen uptake) (Venables et al. 2005; Randell et al. 2016; Fletcher et al. 2017), and the rate can be regarded as the highest systemic capacity to oxidise fat. The exercise intensity, where PFO is reached, is called FAT_MAX_ (Achten et al. 2002). As reviewed by Maunder et al. (2018), the most important determinants of PFO are training status and testing modality, biological sex, as well as habitual and acute nutrition. Large cross-sectional studies have accounted for only 34–47% of the variance in PFO (Venables et al. 2005; Randell et al. 2016; Fletcher et al. 2017). Thus, a large part of the inter-individual variability in PFO remains unexplained. Genetic differences likely play an important role because several physical and performance traits, including maximal oxygen uptake (Bouchard et al. 1998), lean body mass (LBM), muscle strength (Arden and Spector 1997) and skeletal muscle fiber-type proportion (Simoneau and Bouchard 1995) have significant genetic components. Studies investigating the respiratory exchange ratio (RER) at rest and during exercise have demonstrated that the relative use of fatty acids in both conditions show familial resemblance (Bouchard et al. 1989; Toubro et al. 1998). However, to our knowledge, no researcher has studied the absolute fat oxidation rates at rest and during exercise among monozygotic (MZ) twins.

Previous observational studies (Venables et al. 2005; Randell et al. 2016; Fletcher et al. 2017) have highlighted the influence of modifiable lifestyle factors, such as physical activity, on the capacity to oxidise fats. As genes also affect physical activity participation (Stubbe et al. 2006; Mustelin et al. 2012; Aaltonen et al. 2013), observational studies possibly overestimate the influence of physical activity. Experimental studies can provide evidence on the cause-and-effect relationship; however, long-term exercise training trials investigating fat oxidation are rare because they are expensive and arduous to perform. An option to counteract the shortcomings and the difficulties of both study designs is to compare the fat oxidation capacity of MZ co-twins who are discordant in long-term physical activity. This study design controls for genetic predisposition and mostly for the impact of the childhood environment. Therefore, the possible difference between co-twins likely results from different physical activity habits.

Besides investigating the determinants of fat oxidation capacity, researchers have been interested in understanding whether fat oxidation capacity interacts with metabolic health. This seems plausible as efficient utilization of fatty acids could protect from e.g. insulin resistance (Phielix et al. 2012). Indeed, some studies have found an association between systemic fat oxidation and better metabolic health status (Hall et al. 2010; Rosenkilde et al. 2010; Robinson et al. 2015). However, obesity-related increase in fatty acid availability has also been linked to higher fat oxidation levels (Perseghin et al. 2002; Hodson et al. 2010; Ara et al. 2011; Dandanell et al. 2017a). Thus, it remains debated whether higher fat oxidation capacity is beneficial to metabolic health and more research is needed.

In this study, our goal was to investigate the influence of internal (genetics) and external (physical activity) factors on fat oxidation at rest and during exercise. Additionally, we aimed to examine the association between fat oxidation capacity and oral glucose tolerance test (OGTT)-induced metabolic response.

## Materials and methods

### Participants and study design

This study is part of the FITFATTWIN study, whose purpose was to identify possible pairwise differences in health and fitness parameters between male MZ co-twins (aged 32–37 years). The recruitment process was previously reported in detail (Rottensteiner et al. 2015). In short, the studied MZ twin pairs were identified from the longitudinal FinnTwin16 cohort, which follows Finnish twins born from October 1974 to December 1979. The co-twins from 202 male MZ pairs provided data on their physical activities in an online survey, which formed the fifth wave of the FinnTwin16 study data collection. This data was used to identify co-twins who were potentially discordant in leisure-time physical activity (LTPA). From the whole population, 39 twin pairs met the initial selection criteria and were selected to participate in a telephone interview, consisting of questions about their physical activities and health habits. Based on the interview, 20 twin pairs were invited to participate in the study; of these, 17 twin pairs accepted the invitation. Additionally, 6 twin pairs who were identified as concordant in LTPA were recruited from the FinnTwin16 cohort. These pairs were selected to represent varying physical activity levels, from sedentary to athletic. Thus, a total of 23 twin pairs participated in the laboratory measurements performed on 2 consecutive days. The complete timetable of the measurements was reported earlier as supplementary material in Rottensteiner et al. (2015).

Of the 23 twin pairs, 19 pairs participated in the exercise test and 22 pairs participated in the resting metabolism measurement (18 pairs took part in both measurements). One twin individual’s resting metabolism measurement was excluded due to hyperventilation. Thus, the analyses of genetic influence on PFO and RFO were conducted among 19 and 21 twin pairs, respectively. In total, PFO and RFO were determined for 41 and 43 twin individuals, respectively, and the twin individual-based analyses were conducted in these groups. One twin pair declined to participate in the OGTT, and the analyses between PFO or RFO and OGTT variables were performed in groups of 39 and 41 twin individuals, respectively.

Based on detailed LTPA interviews and a questionnaire (see the next subsection), 10 of the 23 twin pairs were identified as LTPA-discordant for the past 3 years. The determination of discordance was thoroughly explained by Rottensteiner et al. (2015). Of the 10 LTPA-discordant twin pairs, 8 pairs participated in both metabolism measurements, and 2 pairs took part in one of the measurements. Therefore, a pairwise comparison on the effect of LTPA on PFO or RFO was performed between 9 twin pairs, respectively.

### Leisure-time physical activity (LTPA)

The LTPA level was determined with two separate interviews and the Baecke questionnaire. A brief retrospective interview (Waller et al. 2008; Leskinen et al. 2009; Rottensteiner et al. 2015), including structured questions on the LTPA’s average frequency, duration, and intensity, as well as the average frequency and duration of commuting, was used to estimate the total LTPA volume at 1-year intervals over the past 6 years. The LTPA volume was calculated by multiplying the activity’s monthly frequency, minute duration and metabolic equivalent of task (MET)-intensity and commuting physical activity was calculated by multiplying the standard 4-MET intensity with the daily commuting minute duration and the weekly frequency (5 times a week). The total LTPA volume was expressed as the sum score of the daily MET-hours, and the mean LTPA (MET-h/day) over the past 3 years (3-year LTMET index) was used to describe each participant’s activity level.

A more thorough interview was used to estimate the past 12-month LTMET index. The interview was based on the Kuopio Ischemic Heart Disease Risk Factor Study Questionnaire (Lakka and Salonen 1997), with additional physical activities. The participants were asked about the number of times per month (and the average duration) they participated in 20 different types of physical activities or other physical activities specified by each respondent. The participants were also asked to classify the intensity of each activity based on a 4-level scale. Like the past 3-year LTMET index, the 12-month LTMET index was calculated as MET-h/day. The participants also completed a 16-item Baecke questionnaire, which measured their recent work, sports and LTPA (Baecke et al. 1982). The total sum score was used for the twin individual-based analysis.

### Peak oxygen uptake (VO_2peak_) and peak fat oxidation (PFO)

A graded incremental exercise test with a gas-exchange analysis was performed on the first day of the laboratory visit. The participants were instructed to avoid vigorous exercise and alcohol use 48 h and avoid eating 2 h prior to testing. The exercise test was performed with an electrically braked bicycle ergometer (Ergoselect 200, Ergoline GmbH, Germany). The testing began with a 2-min stage at 20 W, followed by a 2-min stage at 25 W. Next, the work rate increased by 25 W every 2 min until volitional exhaustion. The breath-by-breath gas exchange was recorded with a Vmax Encore 29 metabolic cart (Sensormedics, Yorba Linda, CA, USA), which was calibrated according to the manufacturer’s instructions before each measurement. The volume of oxygen (VO_2_) inspired and the volume of carbon dioxide (VCO_2_) expired were averaged at 30-s intervals for the whole test duration. The VO_2peak_ was determined as the average of the two highest consecutive VO_2_-measurements. Fat oxidation was calculated for each exercise stage from the last 30-s period with Frayn’s (1983) equation, assuming that the urinary nitrogen excretion was negligible. The highest calculated fat oxidation rate was selected as the PFO and the corresponding exercise intensity as the FAT_MAX_ (%VO_2peak_). Each participant’s heart rate and cardiac function were monitored continuously with a 12-lead electrocardiography system (CardioSoft v.5.02 GE Medical System Corina, GE Medical System Inc., USA). The rating of perceived exertion (RPE) was determined at the end of each stage with the Borg (6–20) scale (Borg 1982). The exercise test was classified as maximal if the RPE was 19–20/20 or the RER was > 1.1 at the end of the test. The exercise test protocol was submaximal for 4 subjects. Among the participants tested with the submaximal protocol, their fat oxidation rates declined before their last performed exercise stage. Thus, their PFO results were included in the study, and their VO_2peak_ was extrapolated based on the submaximal results.

### Body composition

Each participant’s body composition was measured in the morning of the second measurement day, following overnight fasting. For the body mass and height measurements, the participants were barefoot and wore light outfits. Their body mass and height were respectively measured using an electronic scale with a 0.1-kg accuracy and a stadiometer with a 0.5-cm accuracy. Their total mass, LBM, fat mass and body fat percentage were measured with dual-energy x-ray absorptiometry (DXA) (DXA Prodigy, GE Lunar Corp., Madison, WI, USA).

### Resting metabolism

Each participant’s resting metabolism was measured (after the DXA measurement) in a dimly light room. Similar to the exercise test, the same Vmax Encore 29 metabolic cart was used and calibrated accordingly. First, the participants rested 10 min in a supine position. Then, their gas exchange was recorded for 16 min using the ventilated canopy method, and their VO_2_ and VCO_2_ were averaged at 1-min intervals. First 5 min measurement data were excluded. Resting metabolism variables were calculated from a steady-state measurement period (VO_2_ and VCO_2_ coefficient of variation ≤ 10% between minutes). The average steady-state duration was 9.2 ± 2.7 min. The resting energy expenditure (REE) was calculated with the modified Weir equation (Weir 1949; Mansell and Macdonald 1990), and Frayn’s (1983) equation was used to calculate the RFO. A protein correction factor of 0.11 mg/kg/min was applied to take into account the nitrogen exertion (Flatt et al. 1985; Hall et al. 2010; Robinson et al. 2016).

### Metabolic health

A standard 2-h OGTT followed the resting metabolism measurement. After the collection of their fasted blood samples, the participants ingested a 75-g glucose solution (GlucosePro, Comed LLC, Tampere, Finland). Next, their blood samples were collected at 30-min, 1-h and 2-h intervals post-ingestion. All blood samples were collected from each participant’s antecubital vein when he was in a supine position. The plasma glucose concentration was analysed with Konelab 20 XT (Thermo Fisher Scientific, Vantaa, Finland) and the serum insulin concentration was analysed using IMMULITE® 1000 (Siemens Medical Solution Diagnostics, Los Angeles, CA, USA). The Matsuda index was determined based on the equation: 10,000/square root of [(fasting glucose × fasting insulin)  ×  (mean glucose  ×  mean insulin during OGTT)] (Matsuda and DeFrozo 1991). Additionally, the area under the curve (AUC) was calculated for insulin and glucose with the trapezoidal method.

## Ethical approval

Good clinical and scientific practices and guidelines, as well as the Declaration of Helsinki, were followed while conducting the study. The study was approved by the Ethics Committee of the Central Finland Health Care District (Dnro 4U/2011). All participants provided their written informed consent before the laboratory measurements.

### Statistical analysis

Statistical analysis was carried out with IBM SPSS Statistics 24.0 and Stata 15.0. A one-way random model was used to calculate the intraclass correlation coefficients (ICCs) between the MZ co-twins. An ICC compares within-pair variation with between-pair variation and thus explains how similar the co-twins are when compared with the other pairs. Pairwise correlations and differences were analysed with Pearson correlation coefficient and paired-sample *t* test, respectively. Twin individual-based correlations were analysed with simple linear regression, and the within-pair dependency was taken into account (Williams 2000) with the clustering option of Stata. In all regression analyses, RFO or PFO was treated as the dependent variable. All the variables or the regression analysis residuals were determined normally distributed with the Shapiro–Wilk test or with the visual inspection of the histograms and the normality plots. The *p* value 0.05 was selected to represent statistical significance. For clarity, RFO or PFO without a unit symbol is used in the text when the statistical significance persists both when using absolute or LBM relative values in the analysis.

## Results

### Participant characteristics

Table 1 presents the participant characteristics. Overall, the study population consisted of healthy men (aged 32–37 years) with varying physical activity, body composition and cardiorespiratory fitness levels.

**Table 1:** Characteristics of the participants (*n* = 46)

	Mean ± SD	Minimum	Maximum
Age	34.5 ± 1.5	32	37
Body composition			
Height (cm)	178 ± 7	157	190
Body mass (kg)	76.7 ± 9.8	51.5	96.2
BMI (kg/m^2^)	24.1 ± 2.7	19.8	33.6
Lean body mass (kg)	56.8 ± 7.0	40.4	73.2
Fat mass (kg)	16.7 ± 6.7	5.2	31.6
Body fat percentage (%)	21.4 ± 6.7	7.6	36.0
Physical activity			
3-year-LTMET index (MET-h/day)	4.7 ± 4.6	0.2	18.3
12-month-LTMET index (MET-h/day)	4.2 ± 4.6	0.1	27.7
Baecke questionnaire (score)	8.3 ± 1.3	5.6	12.1
Cardiorespiratory fitness^a^			
VO_2peak_ (l/min)	3.2 ± 0.6	2.3	4.6
VO_2peak_ (ml/kg/min)	41 ± 9	29	66
VO_2peak_ (ml/kg LBM/min)	55 ± 8	40	75
Resting metabolism^b^			
REE (kcal/d)	1 685 ± 190	1 297	2 074
RER	0.82 ± 0.04	75	89
RFO (g/min)	0.06 ± 0.02	0.02	0.09
RFO (mg/kg LBM/min)	1.0 ± 0.3	0.5	1.6
Fat oxidation during exercise^a^			
PFO (g/min)	0.39 ± 0.14	0.13	0.81
PFO (mg/kg LBM/min)	6.8 ± 2.2	2.4	13.7
FAT_MAX_ (%VO_2peak_)	40 ± 10	26	72
Metabolic health			
Fasting glucose (mmol/l)	5.5 ± 0.5	4.7	6.6
Fasting insulin (IU/ml)	3.9 ± 3.2	0.2	14.6
2-h OGTT glucose (mmol/l)^c^	5.1 ± 1.1	3.1	7.6
Matsuda index^c^	18.4 ± 17.8	2.3	64.6
Insulin AUC (IU/ml/h)^c^	66.1 ± 38.4	17.6	186.8
Glucose AUC (mmol/l/h)^c^	11.7 ± 2.4	8.2	19.4

*AUC* area under the curve, *BMI* body mass index, *LBM* lean body mass, *MET* metabolic equivalent of task, *OGTT* oral glucose tolerance test, *PFO* peak fat oxidation, *REE* resting energy expenditure, *RER* respiratory exchange ratio, *RFO* resting fat oxidation, *VO*_*2peak*_ peak oxygen uptake

^a^*n* = 41

^b^*n* = 43

^c^*n* = 44 participants

### Hereditary factors and metabolism at rest and during exercise

**Fig. 1:**
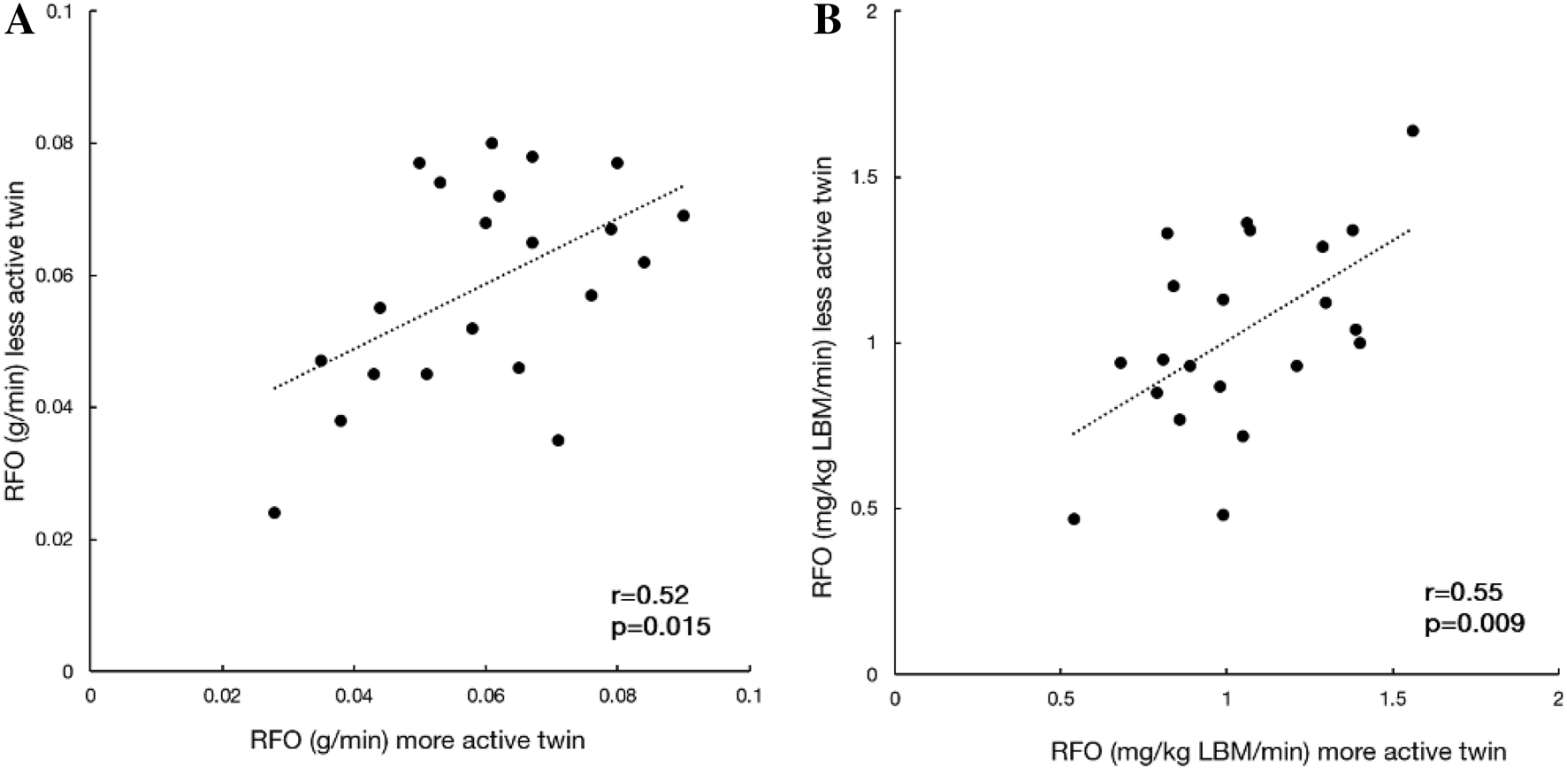
Pairwise correlations of **a** absolute and **b** lean body mass (LBM) relative resting fat oxidation (RFO) in 21 MZ twin pairs

**Fig. 2:**
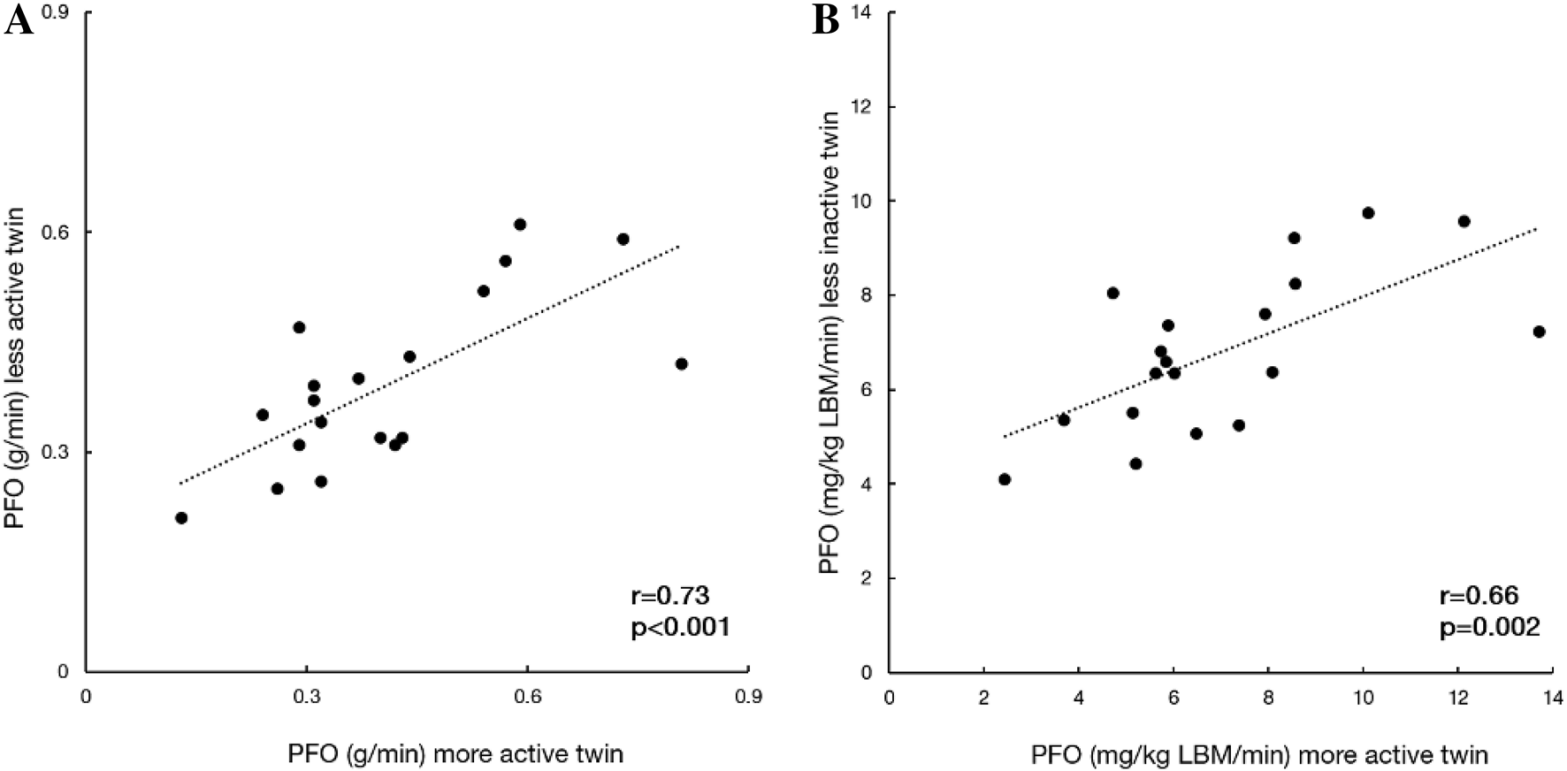
Pairwise correlations of **a** absolute and **b** lean body mass (LBM) relative peak fat oxidation (PFO) during exercise in 19 MZ twin pairs

The calculated ICCs of the resting metabolism variables and PFO showed significant resemblance between co-twins (Table 2). We also categorised the co-twins as more active or less active based on their 12-month LTMET index to calculate pairwise correlations (Figs. 1 and 2). This division did not lead to significant mean differences between the groups in RFO (0.001 g/min, *p* = 0.68) or PFO (0.02 g/min, *p* = 0.47).

**Table 2:** The intraclass correlation coefficients (ICCs) between MZ co-twins

Variable	ICC	95% CI	*p* value
Resting metabolism^a^			
REE (kcal/day)	0.58	(0.21 to 0.80)	0.002
RER	0.51	(0.12 to 0.77)	0.007
RFO (g/min)	0.54	(0.15 to 0.78)	0.004
RFO (mg/kg LBM/min)	0.57	(0.20 to 0.80)	0.003
Fat oxidation during exercise^b^			
PFO (g/min)	0.67	(0.33 to 0.86)	< 0.001
PFO (mg/kg LBM/min)	0.59	(0.21 to 0.82)	0.002
FAT_MAX_ (%VO_2peak_)	0.51	(0.09 to 0.77)	0.010

*PFO* peak fat oxidation, *REE* resting energy expenditure, *RER* respiratory exchange ratio, *RFO* resting fat oxidation

^a^*n* = 21 MZ twin pairs

^b^*n* = 19 MZ twin pairs

### LTPA and metabolism at rest and during exercise

**Fig. 3:**
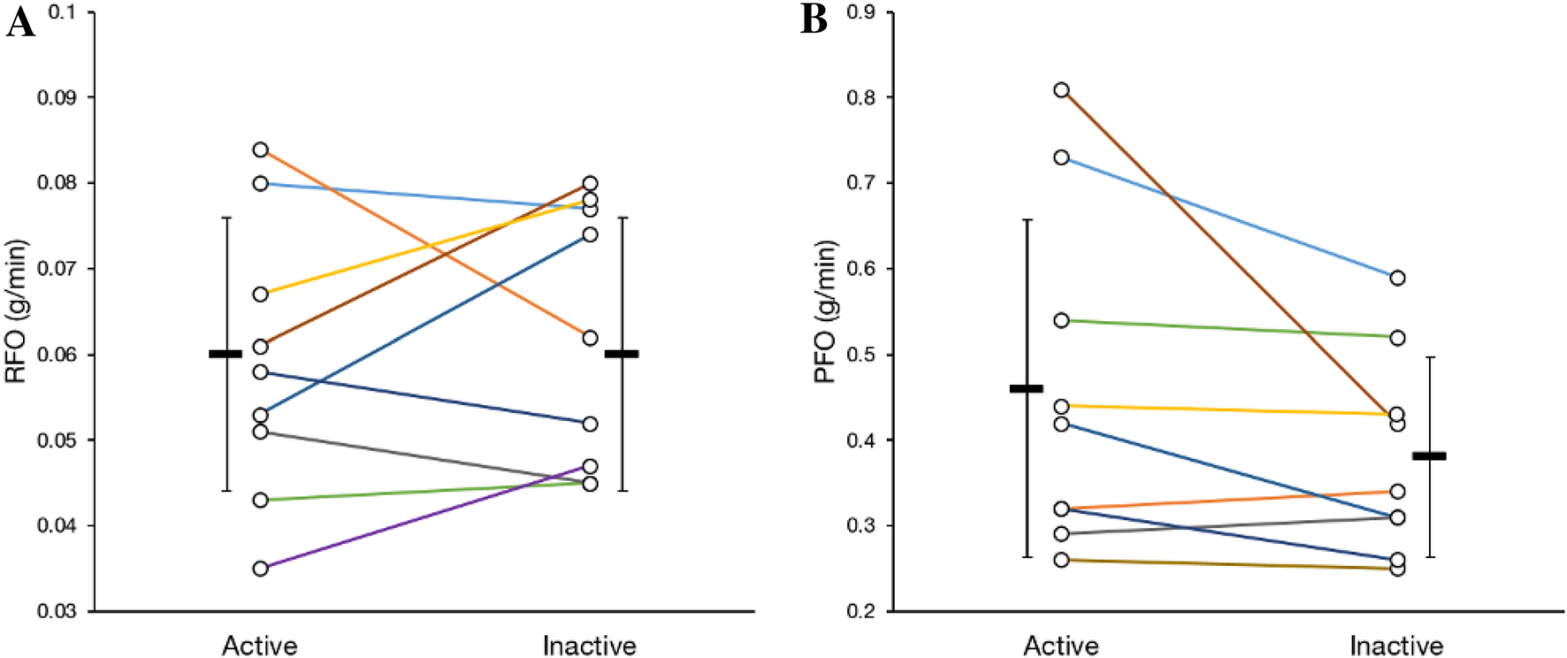
**a** Resting fat oxidation (RFO) and **b** peak fat oxidation (PFO) during exercise in the leisure-time physical activity discordant MZ twin pairs (*n* = 9, 8 pairs successfully participated in both measurements). Figures include group means and standard deviations. Colours represent the same twin pairs in both charts. Note the different scale in the *y*-axis

Table 3 presents the results of the pairwise comparison between the LTPA-discordant co-twins (*n* = 9–10). Figure 3 illustrates individual RFO and PFO results and within-pair relationships. As reported earlier (Rottensteiner et al. 2015), long-term LTPA-discordant co-twins had different body fat percentage and cardiorespiratory fitness levels. However, there were no differences in REE, RER at rest or RFO between active and inactive co-twins. On average, the active co-twins tended to have higher PFO rates and lower FAT_MAX_ when compared with the inactive co-twins, but the differences were not statistically significant. In the twin individual-based analysis (Table 4), only PFO (g/min) was positively correlated with the 12-month LTMET index (*r* = 0.26, *p* = 0.034), the Baecke score (*r* = 0.40, *p* = 0.022) and VO_2peak_ (l/min) (*r* = 0.51, *p* = 0.028).

**Table 3:** Characteristics of the long-term-discordant MZ twin pairs

Variable	Active (*n* = 10)	Inactive (*n* = 10)	Mean difference (95% CI)	*p* value
Physical activity*				
3-year-LTMET index (MET-h/day)	5.0 ± 2.7	1.7 ± 1.3	3.3 (1.9 to 4.8)	0.001
12-month-LTMET index (MET-h/day)	3.9 ± 1.2	1.2 ± 0.9	2.8 (2.0 to 3.5)	< 0.001
Body composition*				
Lean body mass (kg)	56.9 ± 4.8	55.5 ± 6.1	1.4 (− 0.3 to 3.0)	0.094
Fat mass (kg)	16.0 ± 4.5	19.2 ± 6.6	− 3.3 (− 6.7 to 0.2)	0.059
Body fat percentage (%)	20.7 ± 4.0	24.0 ± 4.6	− 3.3 (− 6.2 to − 0.4)	0.029
Cardiorespiratory fitness^a^				
VO_2peak_ (l/min)	3.3 ± 0.3	2.9 ± 0.5	0.4 (0.2 to 0.6)	0.001
VO_2peak_ (ml/kg LBM/min)	58 ± 5	52 ± 5	7 (3 to 10)	0.001
Metabolism at rest^b^				
REE (kcal/day)	1735 ± 187	1675 ± 191	59 (− 48 to 166)	0.24
RER	0.82 ± 0.03	0.80 ± 0.03	0.02 (− 0.01 to 0.04)	0.16
RFO (g/min)	0.06 ± 0.02	0.06 ± 0.02	0.00 (− 0.01 to 0.01)	0.52
RFO (mg/kg LBM/min)	1.0 ± 0.3	1.1 ± 0.3	− 0.1 (− 0.3 to 0.1)	0.35
Fat oxidation during exercise^a^				
PFO (g/min)	0.46 ± 0.20	0.38 ± 0.12	0.08 (− 0.02 to 0.18)	0.11
PFO (mg/kg LBM/min)	8.0 ± 3.1	6.9 ± 1.8	1.1 (− 0.7 to 2.9)	0.18
FAT_MAX_ (%VO_2peak_)	40 ± 9	43 ± 8	− 4 (− 8 to 1)	0.077

*LBM* lean body mass, *MET* metabolic equivalent of task, *OGTT* oral glucose tolerance test, *PFO* peak fat oxidation, *REE* resting energy expenditure, RER: respiratory exchange ratio, *RFO* resting fat oxidation, *VO*_*2peak*_ peak oxygen uptake

^*^Data reported earlier (Rottensteiner et al. 2015)

^a^*n* = 9 MZ twin pairs

^b^*n* = 9 MZ twin pairs, one pair is different than in the exercise test-based variables

**Table 4:** Results of the twin individual-based analysis

	PFO (g/min)	PFO (mg/kg LBM/min)	RFO (g/min)	RFO (mg/kg LBM/min)
Body composition				
BMI (kg/m^2^)	*r* = 0.12 *p* = 0.53	*r* = 0.054 *p* = 0.76	*r* = 0.16 *p* = 0.25	*r* = 0.079 *p* = 0.58
Lean body mass (kg)	*r* = 0.41 *p* = 0.007		*r* = 0.33 *p* = 0.12	
Fat mass (kg)	*r* = − 0.13 *p* = 0.57	*r* = − 0.091 *p* = 0.66	*r* = 0.08 *p* = 0.63	*r* = 0.10 *p* = 0.48
Self-reported leisure time physical activity				
12-month LTMET-index (MET-h/day)	*r* = 0.26 *p* = 0.034	*r* = 0.10 *p* = 0.41	*r* = 0.14 *p* = 0.23	*r* = − 0.08 *p* = 0.46
3-year-LTMET-index (MET-h/day)	*r* = 0.30 *p* = 0.081	*r* = 0.20 *p* = 0.26	*r* = 0.22 *p* = 0.13	*r* = 0.05 *p* = 0.32
Baecke questionnaire (score)	*r* = 0.40 *p* = 0.022	*r* = 0.25 *p* = 0.13	*r* = 0.002 *r* = 0.98	*r* = − 0.15 *p* = 0.24
Cardiorespiratory fitness				
VO_2peak_ (l/min)	*r* = 0.51 *p* = 0.028		*r* = 0.21 *p* = 0.30	
VO_2peak_ (ml/kg LBM/min)		*r* = 0.36 *p* = 0.085		*r* = − 0.006 *p* = 0.97
Fat oxidation during exercise				
PFO (g/min)			*r* = 0.30 *p* = 0.14	
PFO (mg/kg LBM/min)				*r* = 0.29 *p* = 0.12
Glucose homeostasis and insulin sensitivity				
Fasting glucose (mmol/l)	*r* = − 0.11 *p* = 0.42	*r* = − 0.16 *p* = 0.26	*r* = 0.084 *p* = 0.60	*r* = 0.05 *p* = 0.80
Fasting insulin (µU/l)	*r* = − 0.17 *p* = 0.33	*r* = − 0.13 *p* = 0.48	*r* = -0.10 *p* = 0.59	*r* = − 0.079 *p* = 0.63
Matsuda index	*r* = 0.10 *p* = 0.60	*r* = 0.026 *p* = 0.91	*r* = 0.18 *p* = 0.29	*r* = 0.067 *p* = 0.67
Insulin AUC (µU/l/h)	*r* = − 0.42 *p* = 0.015	*r* = − 0.35 *p* = 0.055	*r* = − 0.04 *p* = 0.85	*r* = 0.12 *p* = 0.58
Glucose AUC (mmol/l/h)	*r* = − 0.31 *p* = 0.050	*r* = − 0.27 *p* = 0.11	*r* = 0.21 *p* = 0.082	*r* = 0.31 *p* = 0.019

*AUC* area under the curve, *BMI* body mass index, *LBM* lean body mass, *MET* metabolic equivalent of task, *OGTT* oral glucose tolerance test, *PFO* peak fat oxidation, *REE* resting energy expenditure, *RER* respiratory exchange ratio, *RFO* resting fat oxidation, *VO*_*2peak*_ peak oxygen uptake

### Fat oxidation at rest and during exercise and metabolic health

RFO or PFO were not correlated with fasting glucose, fasting insulin or the Matsuda index in the twin individual-based analysis (Table 4). PFO (g/min) negatively correlated with the AUC of insulin (*r* = − 0.42, *p* = 0.015) and the AUC of glucose (*r* = − 0.31, *p* = 0.050). In contrast, RFO positively correlated (*r* = 0.31, *p* = 0.019) with glucose AUC when expressed relative to LBM.

## Discussion

For the first time, our study data showed that fat oxidation rates at rest and during exercise were similar between MZ co-twins, even though the study group was enriched with pairs who had discordant LTPA habits. The co-twins also exhibited similar FAT_MAX_ values and thus tended to reach PFO at the same absolute exercise intensities. Although we were unable to confirm the effect of long-term LTPA on fat oxidation capacity in our small sub-population of long-term LTPA-discordant MZ co-twins, PFO (g/min) was associated with LTPA in the twin individual-based analysis. We also observed that PFO (g/min), but not RFO, was associated with a favourable response to glucose loading.

This study’s major finding is that hereditary factors influence fat oxidation capacity. The finding supports those of Toubro et al. (1998) and Bouchard et al. (1989), who reported that RER at rest and during low-intensity cycling showed significant familial resemblance. In a study involving male MZ twin pairs (Bouchard et al. 1989), the ICCs of RER ranged from 0.63 to 0.54 during cycling at low intensities (50 W and 100 W, respectively). As the researchers also investigated the substrate use of dizygotic twins, they were able to control their analysis for the common environmental effect. Their calculated heritability estimates ranged from 0.40 to 0.62. However, as RER only describes the relative use of energy substrates, this study broadens the concept by showing that absolute fat oxidation rates behave accordingly and supports the earlier suggestion that genes play a role in determining fat oxidation capacity during exercise (Jeukendrup and Wallis 2005; Randell et al. 2016). This assumption seems evident, as the large cross-sectional studies investigating fat oxidation during exercise have been able to describe only partly the observed inter-individual variability in PFO (Venables et al. 2005; Randell et al. 2016; Fletcher et al. 2017).

We identified a subpopulation of MZ twin pairs, where the co-twins differed in their past 3-year LTPA. As reported earlier (Rottensteiner et al. 2015, 2016; Tarkka et al. 2016; Hautasaari et al. 2017), the LTPA discordance created diet-independent differences between active and inactive co-twins in cardiorespiratory fitness, intra-abdominal adiposity, glucose homeostasis, and brain morphology and function. In this study, we found no differences between the co-twins in their systemic energy metabolism at rest or during exercise. In the twin individual-based analysis, only PFO (g/min) was associated with LTPA. In previous observational studies, PFO was associated with self-reported physical activity (Venables et al. 2005; Fletcher et al. 2017), and trained subjects (Nordby et al. 2006) or athletes (Dandanell et al. 2018) exhibited superior PFO compared with controls. However, it is highly likely that physical activity participation and fat oxidation capacity have shared genetic factors, and the relationship noted in observational studies is partly genetically mediated. In experimental studies, endurance-training interventions commonly increased PFO, at least in untrained populations (reviewed by Maunder et al. 2018). Earlier mechanistic evidence from our laboratory also supports the role of physical activity as a modulator of PFO. In same-sex twin pairs, an over 30-year long physical activity discordance led to significant differences in myocellular gene expression related to oxidative phosphorylation and lipid metabolism (Leskinen et al. 2010). The effects of physical activity on RFO have been investigated less, with mixed results. A modest increase in fat oxidation rates at rest has been reported in some (Barwell et al. 2009; Whyte et al. 2010) but not in all (Scharhag-Rosenberger et al. 2010) trials. When the current scientific evidence is taken together with our results, physical activity seems to be able to influence PFO, while its effect on RFO is questionable.

In the twin individual-based analysis, we observed that PFO (g/min) was associated with a favourable response to glucose loading. The observed inverse association between PFO (g/min) and insulin concentration during the OGTT was especially convincing. However, we found no association between PFO and the Matsuda index, our main surrogate of insulin sensitivity. As explained in the methods section, the Matsuda index is influenced by fasting values, which were not associated with PFO in our study. Previously, Robinson et al. (2015) showed that PFO was inversely associated with a fasting-based QUICKI index. As Robinson et al. (2015) had a larger sample size (*n* = 53) and measured the PFO in the fasting state, they were more able to find the associations between the PFO and the fasting-based values, which generally vary less among healthy individuals when compared with the responses to glucose loading. Here, we show that probably an even more noticeable inverse association exists between PFO (g/min) and the insulin response to the OGTT. However, it should be mentioned that PFO does not always seem to be associated with a healthier metabolic phenotype because an obesity-related increase in fatty acid availability has also been linked to higher PFO (Ara et al. 2011; Dandanell et al. 2017a).

In contrary to PFO, RFO was not associated with a healthy metabolic response to the OGTT. Previous studies have noted mixed findings. Rosenkilde et al. (2010) reported that in a population of overweight but otherwise healthy men, the group with low RER at rest had higher PFO and a healthier metabolic profile when compared with the group with high RER. However, there were no differences in fasting glucose or insulin levels between the groups. Some case–control studies (Perseghin et al. 2002; Hodson et al. 2010) have shown an elevated RFO in obese subjects when compared with their lean counterparts. An elevated RFO could potentially function as a protective mechanism against insulin resistance (Perseghing et al. 2002) and liver fat accumulation (Hodson et al. 2010) when lipid availability increases. Overall, further research is needed to clarify the interaction between systemic fat oxidation and metabolic health.

Our study has both strengths and limitations. A key strength was our ability to measure RFO and PFO in 21 and 19 MZ twin pairs, respectively. This enabled us to investigate the influence of hereditary factors on RFO and PFO in a reasonably sized study group. The calculated ICCs represent the upper bound of heritability, as differences between MZ twins are due to non-genetic factors. However, as MZ twin pairs share also many aspects of their development and environment, the actual heritability of the trait may be lower. A more precise estimation of heritability would require several kinds of relatives (for quantitative trait modeling) or very large study population (for measurement of all genetic variation by whole genome sequencing). Despite our systematic nationwide search, we could only recruit 10 MZ twin pairs, where the co-twins were long-term LTPA-discordant, which weakened our study’s power to find significant pairwise differences. Furthermore, the participants’ MZ twin status made it more difficult to find significant twin individual-based correlations because clustering was necessary to take into account the within-pair dependency. Additionally, since our study included only males, the results cannot be generalised to females.

Another strength of our study was our protocol’s inclusion of an OGTT; we did not depend on using fasting-based values. This enabled us to conduct a more in-depth examination of the possible associations between fat oxidation and metabolic health. However, our study protocol was not optimal for PFO determination, which should be considered when interpreting the results. Nutrition intake the day before (Støa et al. 2016) and on the same day (Achten and Jeukendrup 2003; Edinburgh et al. 2018) will alter substrate use. In this study, we did not control for the nutrition intake before the exercise test. For example, this could partially explain why we did not find any association between RFO and PFO, as previously shown by Robinson et al. (2016). Moreover, we used 2-min exercise stages during PFO testing. The 2-min stages might be too short to reach a steady-state, especially for the subjects with lower cardiorespiratory fitness (Dandanell et al. 2017b; Chrzanowski-Smith et al. 2018). To assess whether the stage duration excessively affected the results, we compared VO_2_ and VCO_2_ between intervals 90–105 s and 105–120 s of the PFO-stage. There were no systematic differences in VO_2_ or VCO_2_ between the intervals. Mean coefficients of variation were 4 ± 4% and 4 ± 4% for VO_2_ and VCO_2_, respectively. Coefficient of variation of VO_2_, VCO_2_ or both exceeded 10% in 3 out of 41 participants. Removing these participants from the analyses did not materially change the results. Therefore, the influence of the stage duration was considered acceptable. Also, the measured PFO (g/min) results were associated with the most important determinants described in the literature, and as expected, correlated between the MZ co-twins. Thus, the measurements seemed to reflect the PFO of our study participants.

In conclusion, we show that fat oxidation rates at rest and during exercise are similar between MZ co-twins. Our results support the suggestion that hereditary factors influence fat oxidation capacity. The internal factors likely set the baseline for fat oxidation capacity that the external factors can modulate. In our study, the role of physical activity seemed smaller, especially concerning RFO. Furthermore, we observed that only higher capacity to utilize fatty acids during exercise associated with better metabolic health.

## Abbreviations

AUC: Area under the curve
DXA: Dual-energy x-ray absorptiometry
ICC: Intraclass correlation coefficient
LBM: Lean body mass
LTPA: Leisure-time physical activity
MET: Metabolic equivalent of task
MZ: Monozygotic
OGTT: Oral glucose tolerance test
PFO: Peak fat oxidation
REE: Resting energy expenditure
RER: Respiratory exchange ratio
RFO: Resting fat oxidation
VCO_2_: Volume of carbon dioxide
VO_2_: Volume of oxygen
VO_2peak_: Peak oxygen uptake
